# Bridging Evolutionary History and Conservation of New World Vultures

**DOI:** 10.3390/ani13203175

**Published:** 2023-10-11

**Authors:** Daniela Cortés-Díaz, Diana L. Buitrago-Torres, Juan Sebastián Restrepo-Cardona, Irene Estellés-Domingo, Pascual López-López

**Affiliations:** 1 Semillero de Investigación en Ecología y Conservación, Universidad de La Salle, Bogotá 110151, Colombia; cortesdd9@gmail.com; 2 Programa de Biología, Universidad del Quindío, Carrera 15 #12N, Quindío 630004, Colombia; dialu1989@gmail.com; 3 Wildlife Ecology and Conservation, University of Florida, Gainesville, FL 32611, USA; jsrestrepoc@gmail.com; 4 Fundación Cóndor Andino—Ecuador, Quito 170143, Ecuador; 5 Movement Ecology Laboratory, Cavanilles Institute of Biodiversity and Evolutionary Biology, University of Valencia, C/Catedrático José Beltrán 2, 46980 Paterna, Valencia, Spain; irene.estelles@uv.es

**Keywords:** extinction, evolutionary distinctiveness, foraging strategies, phylogenetic comparative methods, biogeography, Cathartidae, evolutionary distinctiveness, EDGE, raptors

## Abstract

**Simple Summary:**

This study focuses on New World Vultures, a group of seven scavenger bird species with ecological significance. Despite their importance, there is limited knowledge about their evolutionary history and conservation needs. Recent advances in understanding their evolutionary relationships have paved the way for addressing these gaps using phylogenetic methods. By analyzing the species’ ancestral distribution in the Americas, we employed two techniques to identify historical dispersion patterns. This study pinpointed South America as their original area, with subsequent recolonization of North America by certain species. To guide conservation efforts, we used two indices. The Evolutionary Distinctiveness (ED) index measured species’ uniqueness according to their phylogeny, while the Global Endangerment (GE) index mapped phylogenetic diversity. The findings highlighted the Black Vulture, California Condor and Andean Condor as priority species based on their uniqueness and evolutionary significance. Additionally, we identified crucial regions for conservation, including the lowlands of the Amazon River basin, the Orinoco basin and various areas along the Guiana Shield’s tributaries. This research underscores the importance of combining evolutionary and ecological insights and tools to fill knowledge gaps about species of concern. By doing so, we can formulate effective strategies to protect these species in the face of ongoing biodiversity loss.

**Abstract:**

The New World Vultures (Cathartidae) include seven species of obligate scavengers that, despite their ecological relevance, present critical information gaps around their evolutionary history and conservation. Insights into their phylogenetic relationships in recent years has enabled the addressing of such information gaps through approaches based on phylogeny. We reconstructed the ancestral area in America of the current species using two regionalization schemes and methods: Biogeography with Bayesian Evolutionary Analysis (BioGeoBears) and Bayesian Binary Model–Monte Carlo Markov Chains (BBM–MCMC). Then, we identified the priority species and areas for conservation by means of the Evolutionary Distinctiveness index (ED), as a proxy of the uniqueness of species according to phylogeny, and the Global Endangerment index (GE), mapping phylogenetic diversity. We found that the ancestral area of New World Vultures in America corresponds to South America, with dispersal processes that led to a recolonization of North America by *Coragyps atratus, Gymnogyps californianus* and *Cathartes aura.* We identified the Black Vulture, *G. californianus* and *Vultur gryphus* as priority species based on ED and “Evolutionary Distinct Globally Endangered” (EDGE) indexes, and the lowlands of Amazon River basin and the Orinoco basin and some tributaries areas of the Guiana Shield were identified as the priority areas when mapping the phylogenetic diversity. This study highlights the importance of filling knowledge gaps of species of conservation concern through the integration of evolutionary and ecological information and tools and, thus, developing adequate strategies to enhance the preservation of these species in the face of the current loss of biodiversity.

## 1. Introduction

The New World Vultures (Family: Cathartidae) comprise an emblematic group of raptors that includes seven species of obligate scavenging birds: the Andean Condor (*Vulture gryphus*), Black Vulture (*Coragyps atratus*), California Condor (*Gymnogyps californianus*), Greater yellow-headed Vulture (*Cathartes melambrotus*), King Vulture (*Sarcoramphus papa*), Lesser yellow-headed Vulture (*Cathartes burrovianus*) and Turkey Vulture (*Cathartes aura*) [1,2,3].

Although this family has an undisputed monophyletic status, historically, the phylogenetic position of New World Vultures in avian phylogeny varies among different studies [4,5,6,7,8]. However, more recent studies indicate that Cathartidae may be closely related to the families Accipitridae and Sagittariidae [8,9]. Furthermore, the phylogenetic relationships within the family, mainly between the Andean Condor, California Condor, King Vulture and Black Vulture, have been a focus of discussion. Some analyses with nucleotide sequences show that the Andean Condor is related to the King Vulture, while the Black Vulture is a sister species of the California Condor [9,10,11]. However, Johnson et al. [12] have a different proposal: the family can be divided into two monophyletic groups: in the first one, the Andean Condor, King Vulture and California Condor are related, while in the second one, the Black Vulture is more related to species of the genus Cathartes.

Currently, the New World Vultures are restricted to the Americas and reach their highest diversity in the Neotropical region ([1]; see species’ distribution and IUCN status in http://datazone.birdlife.org/species/search accessed on 10 October 2023). There, the Lesser yellow-headed Vulture is distributed from western Mexico to northern Argentina in areas associated with water bodies, secondary forests and forest edges [13]. The Greater yellow-headed Vulture habits moderately altered and unaltered Amazonian forest and forest–grassland ecotones [13]. The King Vulture is distributed from Mexico to Argentina in dense and open forests, savannahs and coastal meadows [14] and the Andean Condor is distributed throughout the Andes mountain range in primary and secondary forests, “páramos”, open grasslands and coastal areas [15]. In the Nearctic region, the California Condor is restricted to the coastal mountain ranges of Southern California and Northern Baja California in the USA [16]. In contrast, the Black Vulture and the Turkey Vulture are distributed both in the Neotropical and Nearctic regions in a wide variety of both natural and anthropogenic habitats [17].

New World Vultures are among the world’s most threatened birds due to habitat loss and the consequent decrease in food sources as well as the high incidence of poisoning and hunting as a consequence of human–wildlife conflicts [18,19,20,21]. These threats have severely affected populations, mainly of the California Condor and the Andean Condor [22]. Estimates indicate that the California Condor has a global population of less than 500 individuals, so it is categorized as a Critically Endangered species [23], and the Andean Condor has a global population of about 6700 individuals with a declining population size and is classified as Vulnerable [23]. The other New World Vultures are species of Least Concern and wide distribution [24,25,26,27,28]. However, the King Vulture and the Greater yellow-headed Vulture present declining populations [25,28] and trends indicate that New World Vultures could face critical future scenarios if adequate conservation measures are not rapidly implemented [29,30].

The New World Vultures play a critical role within birds’ communities and provide essential ecosystem services, since carrion consumption plays a key role in nutrient flow and mitigates the potential transmission of infectious diseases [31,32,33]. In fact, it has been shown that the decrease in scavengers has caused an increase in the stray dog population and, thus, in human exposure to rabies [34]. In addition, disposal of livestock and human waste by these scavengers has contributed to reducing water pollution [35]. Therefore, these species are able to structure biological communities in ecosystems and are indicators of environmental and human health [36]. Due to their ecological importance and the risk of extinction of some of these species, this group offers an interesting model to evaluate different metrics for the conservation of birds and their habitats.

Thanks to the most recent contributions to the reconstruction of the tree of life in the last two decades (e.g., [37,38,39,40,41], conservation biology has begun to include elements of species evolutionary history in assessments of entire groups of species (see, e.g., https://birdtree.org/; https://earlybird.biology.ufl.edu/, https://b10k.genomics.cn/; all accessed on 10 October 2023). Among these measures, the evolutionary distinctiveness (ED) captures the evolutionary uniqueness of the species, shedding light on more detailed aspects of phylogenetic diversity. Also, when the ED is weighted with the extinction risk, this provides an index known as EDGE, which becomes a useful variable tool in identifying and prioritizing irreplaceable key species in clades and ecosystems [42,43,44] since phylogenetic diversity can also play as a proxy of functional and morphological diversity [45,46,47].

Given their complex evolutionary history, ecological importance and conservation status, the New World Vultures are an interesting group in which to address some information gaps, implementing emerging integrative analyses. Thus, the aims of this study are: (i) to infer the most likely ancestral area of the most common recent ancestor (MCRA) of extant species of New World Vultures; and (ii) to estimate the phylogenetic diversity, evolutionary distinctiveness and EDGE values for this group of birds.

## 2. Methods

**Figure 1:**
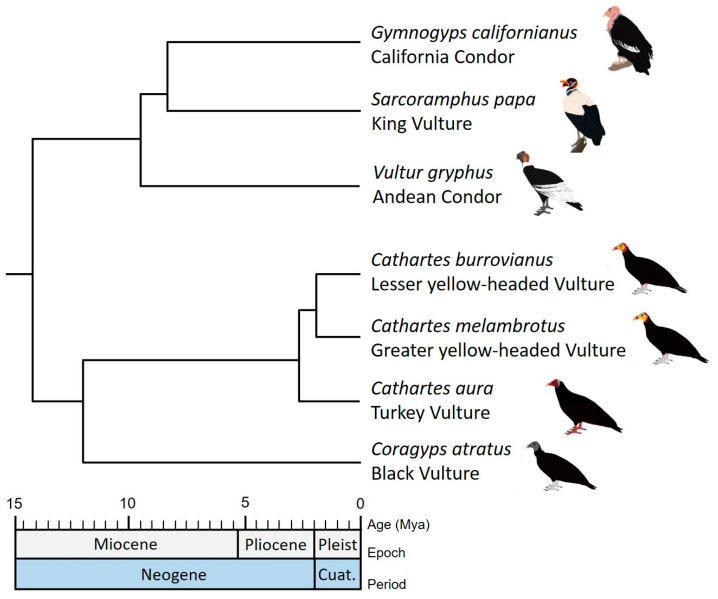
Illustration of the maximum clade credibility tree (MCC) of New World Vultures inferred by Johnson et al. [12].

We used the most complete and robust molecular phylogeny of the New World Vultures inferred by Johnson et al. [12] for all analyses in this study. This phylogeny corresponds to a maximum clade credibility tree (MCC), reconstructed with Bayesian inference from two mtDNA genes (Cyt-b and ND2) and five sets of nuclear introns (EEF2, GAPDH, HMGN2, RHOD and TGFb2). Johnson’s [12] phylogenetic tree provides an estimate of divergence times and is supported by posterior probabilities of nodes mostly greater than 0.8. It includes representative species of the most related families (e.g., Pandionidae, Sagittariidae and Accipitridae). However, we keep apart the clade corresponding to the New World Vultures species in order to perform the subsequent analyses (Figure 1).

### 2.1. Biogeography

We used a sample of 151 trees by Johnson et al. [12] sourced from a GitHub repository (https://github.com/FePhyFoFum/phyx accessed on 10 October 2023) in combination with the MCC. For the inference of the MRCA of the New World Vultures, we performed biogeographic reconstructions by setting two large-scale regionalization schemes: a first biogeographic reconstruction in which we used the Neotropical and Nearctic regions and a second biogeographic reconstruction in which we used three more specific areas, namely (i) North America (including the United States and northern Mexico), (ii) Central America (from the tropical belt of Mexico to the Isthmus of Panama) and (iii) South America. We did not increase the spatial resolution of the analyses to include more areas, since the probabilities of the ancestral areas calculated by the algorithm (<15%) were very low relative to the uncertainty (20%).

We tested two methods implemented in Reconstruct Ancestral State in Phylogenies (RASP): Biogeography with Bayesian Evolutionary Analysis (*BioGeoBears*) and Bayesian Binary Model–Monte Carlo Markov Chains (BBM–MCMC) [48]. *BioGeoBears* is an R package incorporated in RASP that allows one to infer ancestral areas by probabilistically comparing several models, namely Dispersal–Extinction–Cladogenesis (DEC), a likelihood interpretation of Statistical Dispersal–Vicariance Analysis (DIVALIKE), a likelihood interpretation of the Bayesian inference of historical biogeography for many discrete areas method (BAYAREALIKE) and a version of each of these models including the “J” parameter [49]. We computed the Akaike Information Criterion corrected for small samples (AICc) for model ranking. The BBM explains the phylogenetic uncertainty when estimating the probability of an ancestral range in an averaged node on a posterior set of trees [50]. In this analysis, we implemented the F81 Gamma model and ran 10 MCMC for 1,000,000 generations, sampling every 100 generations, with 25% of the initial samples discarded as burn-in [50,51]. In BBM analysis, the maximum number of areas in both biogeographical reconstructions was set to one, in order to override combinations of areas, so that the probability of each individual area on the nodes could be determined.

### 2.2. Phylogenetic Diversity, Evolutionary Distinctiveness and EDGE Species

The identification of EDGE species requires the analysis of two components: Evolutionary Distinctiveness (ED) and Globally Endangered (GE) scores, based on the IUCN Red List categories. To this end, firstly, we calculated the Evolutionary Distinctiveness (ED) by “Fair proportion” of each species using the *evol.distinc* function implemented in the *picante* R package [52]. The “Fair proportion” measure consists of the sum of branch lengths from root to each tip divided by the number of species descending from each branch [53]. Then, to obtain the Globally Endangered (GE) scores, we turned the current IUCN Red List categories of species [23] into numerical values as proposed by Isaac et al. [42] as follows: Least Concern (LC) = 0, Near Threatened (NT) and Conservation Dependent (LR/cd) = 1, Vulnerable (VU) = 2, Endangered (EN) = 3 and Critically Endangered (CR) = 4. With the ED and GE values, we identified the EDGE species by applying the following formula proposed by Isaac et al. [42]: EDGE = ln (1 + ED) + (GE × ln (2)). To optimize these calculations, we compiled this formula into a function called *edge.species* (S1).

In order to quantify the loss of the phylogenetic diversity of New World Vultures, we tested for hypothetical scenarios of extinction of species that reached the highest ED and EDGE values. First, we calculated the phylogenetic diversity on the phylogeny of the whole clade with the *pd* function in the *picante* R package [52]. This function computes the total sum of phylogenetic branch lengths for any given dataset with an associated phylogeny. It should be noted that phylogenetic diversity (PD) is not statistically independent of species richness but rather shows a positive correlation with species richness. Then, we performed the same calculation on phylogenetic trees excluding each of the identified priority species. Finally, we mapped the phylogenetic diversity of New World Vultures to identify the areas of highest biodiversity score in terms of each group’s evolutionary history using the distribution polygons of each species from IUCN spatial data and mapping [23] and the *phyloregion* R package [54]. We converted the polygons into a community matrix using the *polys2comm* function with a resolution of 1 × 1 degree grid cells. Then, we calculated the phylogenetic diversity with the *pd* function and, finally, we projected the results onto a map of America using the *plot_swatch* function. In both analyses, phylogenetic diversity was calculated as the total length of all branches of a set of taxa in a phylogenetic tree [55].

## 3. Results

### 3.1. Biogeography

**Figure 2:**
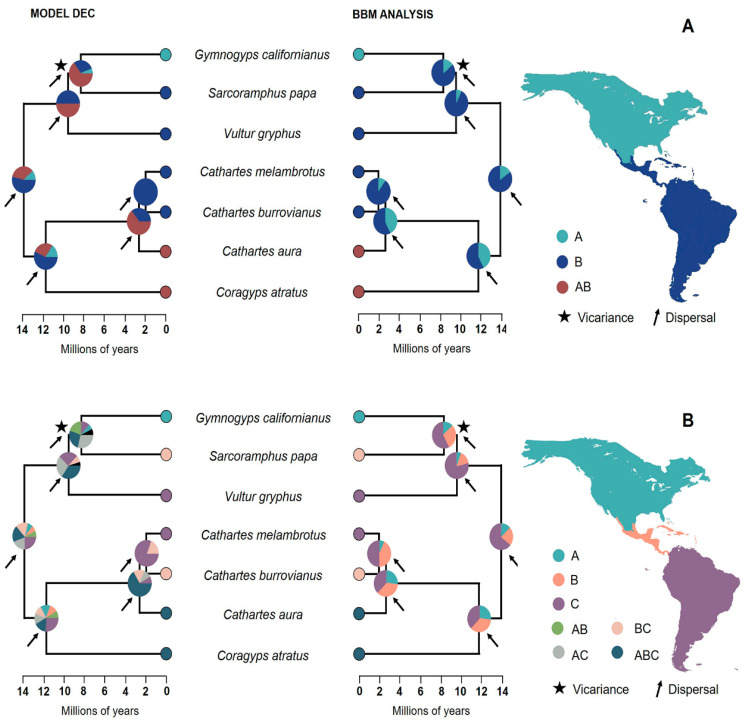
Reconstruction of the ancestral areas inferred from the BBM analysis and the DEC model using two regionalization schemes: (**A**) Nearctic and Neotropical region; (**B**) North America, Central America and South America. The pie charts in the nodes represent the marginal probabilities of each area and the areas combined. The biogeographic events associated with the diversification and distributions of the seven species of New World Vultures in America are indicated by arrows and asterisks. The letters in each reconstruction refer to the chosen areas; in the reconstruction of the first regionalization scheme (above), we have the Nearctic area (A), the Neotropical area (B) and the combination of both areas (AB), while in the reconstruction of the second regionalization scheme (below), we have the area of North America (A), the area of Central America (B and area of South America (C) and the rest of the letters are the combination of the main areas mentioned.

According to model ranking, the *BioGeoBears* model that best fits the data in both regionalization schemes is DEC (Table 1 and Table 2). Reconstruction with this model suggested that the most likely ancestral area of the MCRA of extant New World Vulture species was the Neotropical region, particularly South America (Figure 2A,B). Although ancestral area estimation using the DEC model did not provide high resolution, the exclusion of combined areas in BBM analysis supports with a high probability (>75%) a neotropical–South American origin (Figure 2A,B). Reconstruction of ancestral areas indicated that dispersal was a key element in the speciation processes that shaped the current distribution patterns of New World Vultures in America. Our results suggest that a first dispersal event in the neotropics during the Miocene split the ancestral lineage into the clade of vultures (Black Vulture, Turkey Vulture, Lesser yellow-headed Vulture and Greater yellow-headed Vulture) and the clade of the condors (California Condor, King Vulture and Andean Condor). Later, during this same period, a second dispersal event triggered the divergence of the genus *Cathartes* and the Black Vulture. The Andean Condor then separated from the California Condor and King Vulture lineage by a third process of dispersal that allowed the former species to colonize the Andes. The divergence of the California Condor and the King Vulture has a dispersion component, which probably promoted the establishment of the California Condor in the Nearctic region, and a vicariant one, which is reflected in the disjunction between the current distributions of both species.

### 3.2. Phylogenetic Diversity, ED and EDGE Species

**Figure 3:**
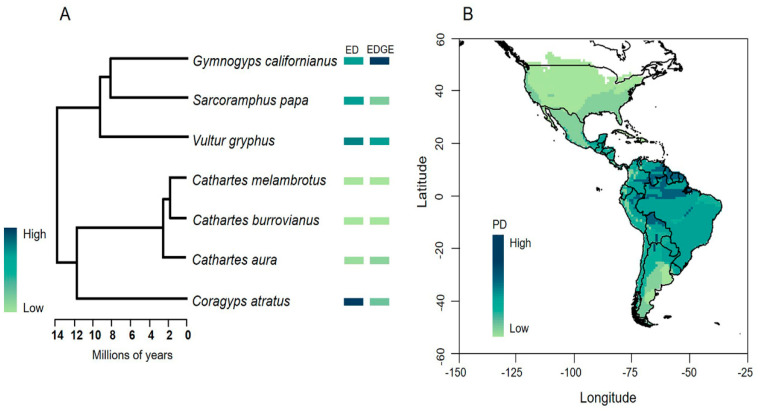
(**A**) Evolutionary distinctiveness (ED) and Evolutionary Distinct and Globally Endangered (EDGE) scores. (**B**) Map of phylogenetic diversity (PD) of New World Vultures in America based on distribution maps of species according to the IUCN. Geographic coordinates are expressed in decimal degrees.

The mean evolutionary distinctiveness (ED) score for New World Vulture species was 8.83 Ma, with the Black Vulture (ED—12.32 Ma) and the Andean Condor (ED—10.90 Ma) reaching the highest values (Figure 3A). Linking ED scores to GE, the two species with the highest risk of extinction were the California Condor (CR) and the Andean Condor (VU). Overall, this indicates that the Black Vulture, California Condor and Andean Condor are priority species for conservation, given their distinctive evolutionary history (Figure 3A). The phylogenetic diversity (PD) for New World Vulture species was 62 Ma. When comparing the three hypothetical scenarios of extinction of the ED and EDGE species, the magnitude of the loss of phylogenetic diversity given the extinction of the Black Vulture, Andean Condor and California Condor would be 19%, 16% and 13%, respectively. Finally, by mapping the phylogenetic diversity of New World Vultures, we found that the greatest richness and phylogenetic diversity of this group of species are located in the Neotropics and tend to decrease towards temperate areas. In fact, the maximum values are concentrated in the Amazon, mainly in the lowlands close to the Amazon basin and the Orinoco basin and other tributaries of the Guiana Shield (Figure 3B). In this area, five of the seven species are distributed: the Black Vulture, Turkey Vulture, Lesser yellow-headed Vulture, Greater yellow-headed Vulture and King Vulture, which together represent 44.2 Ma of evolutionary history of the New World Vultures.

## 4. Discussion

Multiple hypotheses about the center of origin of the New World Vultures have arisen. On the one hand, a North American origin was proposed [56] based on a fossil from the late Eocene recorded in the United States [57] and, on the other hand, fossil records in Europe dated from the transition Eocene/Oligocene support an Old World origin [7,58]. Thus, this issue remains the focus of a controversial debate that is still in place and requires more fossil evidence to be solved. In spite of this, advances in molecular phylogenetics allow us to account for the dated phylogeny of the current species of New World Vultures, and with this resource now available, we are able to infer the most recent biogeographic history of the group, that is, from its arrival into the New World.

Our results suggest that the radiation of the seven current species of New World Vultures took place in South America (Figure 2A,B). This hypothesis is supported by the record of a Brazilian fossil morphologically similar to *Coragyps* dated from Late Oligocene/Early Miocene [59,60] that is considered the oldest and best documented fossil of the family in New World [61]. Then, two major events took place during the Middle Miocene: on the one hand, speciation processes within the Condor clade gave rise to the Andean Condor that colonized the Andes ~9 Ma ago [12], a period that coincides with the period after the rampant orogenic processes of the region during the Early–-Middle Miocene (as mentioned by Blandin and Purser [62] and references therein). On the other hand, dispersal events allowed the colonization of North America by the Black Vulture and the California Condor. After their arrival, it is presumed that the genus *Gymnogyps* reached a wide distribution, even outside North America, during the Pleistocene [63]. Regarding the whole clade of the condors, based on two fossils assigned to this group recorded in the United States dated ~ 15–13 Ma, it has been suggested that the clade originated in North America and radiated in South America [60] as a result of a dispersal process facilitated by the coastal winds of the Western Andes [64]. Although this idea has been sustained for years, according to our results, there is no likely reason to consider North America as the ancestral area of the clade of condors, since this hypothesis implies a second recolonization event of South America by the King Vulture and the Andean Condor, a less cost-effective and parsimonious process than a South-American radiation with a single recolonization northward. The genus *Cathartes* radiated during the Pliocene in South America, and then a process of expansion of the distribution of the Turkey Vulture could have allowed this species to reach North America.

It has been suggested that the radiation of megafauna in South America after the Middle Miocene and the consequent increase in carrion availability [65,66] are related to the diversification of scavengers in the New World. In particular, the increase in species richness of the New World Vultures in the Plio-Pleistocene is strongly supported by numerous fossil records in several localities across South America [61,67,68,69,70,71,72,73,74,75]. In consequence, a presumable increase in interspecific competition could have triggered the development of distinctive traits and behaviors to optimize the exploitation of resources, as the differential capacity in the olfactory system [76,77,78] thus, decreasing the overlap between sympatric species [77,78].

Our findings indicate that the Black Vulture, Andean Condor and California Condor must be considered conservation-priority species based on their uniqueness and evolutionary significance (Figure 3A). The Black Vulture is a resilient species that adapts to different types of habitats [79] and is listed as a species of Least Concern according to IUCN criteria [27]. Studies have shown that in some geographic areas, Black Vultures are constantly threatened by high levels of lead contamination and conflicts over wildlife that could be detrimental to their populations [20,80,81]. Species conventionally considered at low risk of extinction and marginalized from conservation plans can reach high ED values, so traditional prioritization systems could be masking their real importance [44]. In this sense, we highlight the importance of increasing efforts for the conservation of the Black Vulture, since its extinction would represent the loss of a significant amount of unique evolutionary history (~12 Ma), corresponding to 19% of the total phylogenetic diversity of the family.

Due to anthropogenic threats such as habitat degradation, poisoning with pesticides, lead intoxication, illegal capture, free-ranging dogs and shooting [21,82,83,84], Andean Condors and California Condors, listed as Vulnerable and Critically Endangered [23], respectively, have suffered considerable population declines and geographic range contractions [85,86,87,88]. Furthermore, it is also expected that climate change will cause Black Vultures to move to higher altitudes and this will consequently increase overlap and competition with Andean Condors [89]. In this scenario, the risk of extinction of the Andean Condor would increase, since its populations are competitively excluded by Black Vultures [90,91]. It is fundamental to develop conservation strategies focused on Andean Condors and California Condors, as well as on Black Vultures [84,92]. The protection of these three species could allow the preservation of unique phenotypic and ecological traits that may lead to more stable biological systems [93,94]. If adequate management actions are not taken, the ecological, economic and evolutionary impact generated by the reduction in the geographic range and the subsequent extinction of scavengers will be critical [34,42,95,96]. In order to reduce anthropogenic threats on New World Vultures and their direct consequences, the selection of strategic areas for conservation, an approach that has also been suggested for groups of scavengers in the Old World (see [97]), becomes essential.

We emphasize the Amazon basin as a key area for the protection of ~70% of the phylogenetic diversity of New World Vultures (Figure 3B). The Amazon basin is one of the main sources of biodiversity, mainly due to the major speciation events that have taken place since the Cenozoic, giving rise to several Neotropical lineages [98]. Several areas of the Amazon region have been highlighted as priorities for the conservation of evolutionary history because they host high levels of phylogenetic diversity of different taxonomic groups such as lizards, snakes, turtles and plants [99,100,101]. Faced with the global crisis of biodiversity loss, identifying phylogenetic diversity hotspots, as a result of the integration of spatial data and evolutionary analysis, is an ideal proxy for the conservation of genetic diversity, an aspect that has become crucial for international cooperation initiatives such as the Aichi Biodiversity Targets established by The Convention on Biological Diversity (CBD) and the UN Sustainable Development Goals (SDGs).

## 5. Conclusions

In this study, we underscore the critical significance of harnessing available resources to employ a diverse array of analytical phylogenetic methods. These approaches serve as powerful tools not only for bridging information gaps but also for advancing our understanding not just of the evolutionary trajectories of organisms but also of their conservation and management strategies. It is essential, however, to acknowledge and address certain inherent limitations within this study. One such limitation stems from the use of a phylogeny constructed based on a restricted set of genes. Additionally, the exclusion of closely related extinct taxa from our phylogeny is, regrettably, a consequence of the limited availability of suitable information. These limitations, namely the reliance on a gene-limited phylogeny and the omission of extinct taxa due to data constraints, inevitably constrain the resolution of our analytical approaches and, consequently, the depth of our inferences.

In conclusion, our research not only highlights the invaluable role of comprehensive phylogenetic investigations but also underscores the need for continued efforts to address these limitations. For groups of species that have received minimal scientific attention, we advocate for dedicated research endeavors aimed at reconstructing dated molecular phylogenies that encompass all relevant species. By doing so, we can potentially illuminate previously uncharted facets of their biology, offering invaluable insights derived from a more comprehensive understanding of their evolutionary relationships.

## Figures and Tables

**Table 1:** Comparison of the six models evaluated with BioGeoBears for the regionalization scheme of two areas (Neotropical and Nearctic) and their respective parameters and scores: Dispersion (d), Extinction (e), Founder (j) and Akaike information criterion corrected for sample size (AICc).

Rank	Model	Parameters	d	e	J	AICc
1	DEC	2	0.071	0.0084	0	22.07
2	DIVALIKE	2	0.084	0.0062	0	22.72
3	BAYAREALIKE	2	0.081	0.039	0	24.91
4	DEC + J	3	0.044	1.0 × 10^−12^	0.38	26.49
5	DIVALIKE + J	3	0.056	1.0 × 10^−12^	0.36	27.49
6	BAYAREALIKE + J	3	0.037	1.0 × 10^−7^	0.29	29.89

**Table 2:** Comparison of the six models evaluated with BioGeoBears for the regionalization scheme of three areas (North America, Central America and South America) and their respective parameters and scores: Dispersion (d), Extinction (e), Founder (j) and Akaike information criterion corrected for sample size (AICc).

Rank	Model	Parameters	d	e	J	AICc
1	DEC	2	0.078	0.0089	0	31.73
2	DIVALIKE	2	0.088	1.0 × 10^−12^	0	31.93
3	BAYAREALIKE	2	0.10	0.078	0	33.76
4	DEC + J	3	0.061	1.0 × 10^−12^	0.56	36.6
5	DIVALIKE + J	3	0.071	1.0 × 10^−12^	0.50	37.57
6	BAYAREALIKE + J	3	0.011	0.079	1.0 × 10^−5^	40.76

## Data Availability

The data on the molecular phylogeny of the New World Vultures inferred by Johnson et al. [12] are publicly available at the GitHub repository (https://github.com/FePhyFoFum/phyx, accessed on 10 October 2023).
